# Programmed cell death in *Acanthamoeba castellanii* Neff induced by several molecules present in olive leaf extracts

**DOI:** 10.1371/journal.pone.0183795

**Published:** 2017-08-31

**Authors:** Ines Sifaoui, Atteneri López-Arencibia, Carmen Mª. Martín-Navarro, María Reyes-Batlle, Carolina Wagner, Olfa Chiboub, Mondher Mejri, Basilio Valladares, Manef Abderrabba, José E. Piñero, Jacob Lorenzo-Morales

**Affiliations:** 1 Laboratoire Matériaux-Molécules et Applications, IPEST, Institut Préparatoire aux Etudes Scientifiques et Techniques, La Marsa, University of Carthage, Tunis, Tunisia; 2 University Institute of Tropical Diseases and Public Health, University of La Laguna, Avda Francisco Sanchez s/n, Campus de Anchieta, la Laguna Tenerife, Canary Islands, Spain; 3 Cátedra de Parasitología, Escuela de Bioanálisis, Facultad de Medicina, Universidad Central de Venezuela, Caracas, Venezuela; Albert Einstein College of Medicine, UNITED STATES

## Abstract

Therapy against *Acanthamoeba* infections such as Granulomatous Amoebic Encephalitis (GAE) and *Acanthamoeba* Keratitis (AK), remains as an issue to be solved due to the existence of a cyst stage which is highly resistant to most chemical and physical agents. Recently, the activity of Olive Leaf Extracts (OLE) was demonstrated against *Acanthamoeba* species. However, the molecules involved in this activity were not identified and/or evaluated. Therefore, the aim of this study was to evaluate the activity of the main molecules which are present in OLE and secondly to study their mechanism of action in *Acanthamoeba*. Among the tested molecules, the observed activities ranged from an IC_50_ of 6.59 in the case of apigenine to an IC_50_ > 100 μg/ml for other molecules. After that, elucidation of the mechanism of action of these molecules was evaluated by the detection of changes in the phosphatidylserine (PS) exposure, the permeability of the plasma membrane, the mitochondrial membrane potential and the ATP levels in the treated cells. Vanillic, syringic and ursolic acids induced the higher permeabilization of the plasma membrane. Nevertheless, the mitochondrial membrane was altered by all tested molecules which were also able to decrease the ATP levels to less than 50% in IC_90_ treated cells after 24 h. Therefore, all the molecules tested in this study could be considered as a future therapeutic alternative against *Acanthamoeba* spp. Further studies are needed in order to establish the true potential of these molecules against these emerging opportunistic pathogenic protozoa.

## Introduction

Free living amoebae of *Acanthamoeba* genus are ubiquitous microorganisms which inhabit several niches such as air, soil, water, contact lenses, air conditioning units and other environments. Moreover, these amoebae are able to cause human infections such as Granulomatous Amoebic Encephalitis (GAE) and *Acanthamoeba* Keratitis (AK) [1–3]. Current therapeutic approaches are compromised by resistance, variable efficacy between strains or species and side and toxic effects.

The Olive tree has been used by native people from the Mediterranean basin to treat different diseases such as malaria and ulcers. Phytochemical investigations of this plant correlate its benefits to its richness on bioactive molecules including phenolic compounds and triterpenic acids among others [4, 5].

Phenolic compounds or polyphenols are considered as the most important secondary metabolites produced by plants. Indeed, these molecules are present in different parts of the plant and their quantity as well as profiles depends strongly on the nature of the plant organ, variety, location, climate, etc. Biophenols could be subdivided in simple phenols, phenolic acids (benzoic acid, cinnamic acid and its derivatives), flavonoids (flavonols, flavonones, isoflavone, anthocyanins, etc.), lignans, stilbenes and tannins. Oleuropein and its related derivatives (hydroxytyrosol and Tyrosol) constitute the major phenolic compounds present in OLE. Several studies have reported the antioxidant, hypoglycemic, antihypertensive, antimicrobial, antitumoral, antiatherosclerotic, antiparasitic and antiviral (including anti-HIV), properties of these compounds [6–9]. Flavones (luteolin, apigenin and their derivatives) constitute the major class of flavonoids present in OLE [10]. Several authors have reported their therapeutic effects against many diseases such as cancer, cardiovascular and neurological disorders. Triterpenic acids as oleanolic, ursolic; maslinic and pentacyclic triterpenes, are widely present in plants and especially in olive trees. These compounds have attracted much interest due to their biological activities such as anti-viral anti-diabetes and anti-tumoural properties [11–13].

Our preliminary work with OLE from Tunisian origin showed that the ethyl acetate fraction presented high activity against the trophozoïte stage of *Acanthamoeba* spp [4]. Thus, in this work it was aimed to evaluate the activity of the major molecules present in OLE and secondly to elucidate key factors involved in the mechanism of action of these molecules against *Acanthamoeba* spp.

## Material and methods

### Molecules/Chemicals

Twenty four molecules were used to evaluate their *in vitro* activity against *Acanthamoeba castellanii* Neff. Gallic acid, vanillin, caffeic acid, ferulic acid, chlorogenic acid, *p*-coumaric acid, *m*-coumaric acid, ellagic acid, vanillic acid, syringic acid, tyrosol, protochatechuic acid, rutin, catechin oleuropein and hydroxytyrosol were purchased from Sigma Aldrich (Tres Cantos, Madrid, Spain), the luteolin, lueolin-7- O -glucoside, apigenine, versbascoside, quercetin and the ursolic acid were purchased from Extrasynthese (Cymit quimica, Barcelona, Spain), as for the oleanolic and maslinic acids they were isolated and purified from olive leaf extraction accordingly to Sifaoui et al, (2014a) [5]. Stock solutions have been prepared by dissolving the molecules in the dimethyl sulfoxide (DMSO; Sigma Aldrich (Tres Cantos, Madrid, Spain) at a concentration of 10 mg/ml.

### *In vitro* sensitivity and activity assays

#### *Acanthamoeba* strains

The strains used in this study included a type strain: *Acanthamoeba castellanii* Neff (ATCC 30010), and two clinical isolates, CLC-16, genotype T3; and CLC-51, genotype T1, which were isolated in a previous study [14]. The strains were axenically grown in PYG medium (0.75% (w/v) proteose peptone, 0.75% (w/v) yeast extract and 1.5% (w/v) glucose) containing 40 μg/ml of gentamicin (Biochrom AG, Cultek, Granollers, Barcelona, Spain).

#### *In vitro* effect against the trophozoite stage of *Acanthamoeba*

The anti-*Acanthamoeba* activities of the tested molecules were determined using the Alamar Blue^®^ assay as previously described [14, 15]. Briefly, *Acanthamoeba* strains were seeded in duplicate on a 96-well microtiter plates with 50μl from a stock solution of 10^4^ cells/ml. Amoebae were left to adhere for 15 min process which was checked using a Leika DMIL inverted microscope (Leika, Wetzlar, Germany). After that, 50 μl of serial dilutions of the molecules to be evaluated were added to the wells (In all tests, 1% DMSO was used to dissolve the highest dose of the compounds without inducing any effects on the parasites). Finally the Alamar Blue Reagent^®^ (Life Technologies, Madrid, Spain) was placed into each well at an amount equal to 10% of the final volume. Test plates containing Alamar Blue were then incubated for 120 h at 28°C with a slight agitation.

Subsequently the plates were analyzed, during an interval of time between 72 and 144 h, on an EnSpire^®^ Multimode Plate Reader (Perkin Elmer, Madrid, Spain) using a test wavelength of 570 nm and a reference wavelength of 630 nm. Percentages of growth inhibition, 50% and 90% inhibitory concentrations (IC_50_ and IC_90_) were calculated by linear regression analysis with 95% confidence limits. All experiments were performed three times, and the mean values were also calculated.

### Cytotoxicity test

A commercial kit was used for the evaluation of the induced cytotoxic effects of tested compounds based on the measurement of lactate dehydrogenase (LDH) quantity released to the media (LDH Cytotoxicity Detection Kit, Roche Applied Science, Madrid, Spain), following the manufacturer's instructions. Briefly, the macrophages J774.A1 (ATCC # TIB-67) were incubated with different concentrations of the tested compounds for 24 hours in duplicate. After incubation, supernatants were obtained and LDH levels were determined following manufacturer′s instructions. To determine the cytotoxicity percentages, the average absorbance values of the duplicates were calculated and compared with negative and positive controls. Cytotoxic levels were determined as previously described [16].

### Cysticidal activity

The effects of the active molecules against cysts were evaluated by incubating 10^4^ cysts of *A*. *castellanii* Neff with the previously calculated IC_90s_ of the bioactive molecules in PYG medium. The numbers of trophozoïtes, cysts, and nonviable cysts were visually counted with a Neubauer chamber at 96, 120, 144, and 168 h using an inverted microscopy.

### Image-based cytometry analysis for apoptosis determination

Annexin-V/propidium iodide (PI) double staining assay was performed using the Tali^®^ Apoptosis Kit—Annexin V Alexa Fluor^®^ 488 & Propidium Iodide according to the manufacturer's instructions (Life Technologies, Madrid, Spain). Briefly, after being treated with IC_50_ and IC_90_ of the tested molecules for 24 h, amoebae were centrifuged at 250 g for 10 minutes, washed twice with the Annexin Binding Buffer (ABB) and incubated with 5 μl of annexin-V for 20 min. After that, cells were centrifuged and resuspended in ABB containing 1 μl of PI and incubated for 3 min at room temperature. Finally, 25 μl of the stained cells were loaded into a Tali^®^ Cellular Analysis Slide and were analysed in the Tali^®^ Image-Based Cytometer. Data were collected using the Tali^®^ data acquisition and analysis software (Life Technologies, Madrid, Spain) [17].

### Plasma membrane permeability

The SYTOX^®^ Green assay was performed to detect membrane permeability alterations on the parasites. Briefly, 10^5^ amoebae/ml were incubated with the SYTOX^®^ Green at a final concentration of 1 μM (Molecular Probes^®^) for 30 min in the dark at 26°C. Subsequently, parasites were disposed to black plates and the tested compounds were added at the IC_90_. The increase in fluorescence due to binding of the fluorescent marker to the amoebic DNA was measured using an EnSpire^®^ Multimode Plate Reader (Perkin Elmer, Madrid, Spain) with excitation wavelength of 504 nm and emission wavelength at 523 nm, and expressed as percentage relative to full permeabilized cells achieved by the addition of 0.1% Triton X-100 [17–19].

### Changes in the mitochondrial membrane potential (ΔΨm)

The ΔΨm was measured using JC-1 Mitochondrial Membrane Potential Assay Kit (Cayman Chemical, Vitro, Madrid, Spain). This lipophilic cationic probe accumulates in the mitochondrial matrix according to the membrane potential. In healthy cells with a high ΔΨm, JC-1 spontaneously forms complexes known as J-aggregates, showing intense red fluorescence (emission at 595 nm). In apoptotic or unhealthy cells with a low ΔΨm, JC-1 remains in its monomeric cytosolic form and shows only green fluorescence (emission at 535 nm). In brief, amoebae, after 24 hours of incubation with different concentrations of the tested compounds, were harvested and washed with buffer. The cells were then incubated at 26°C for 30 minutes with JC-1 dye. Cells were then analyzed by fluorescence measurement in black plates through spectrofluorometer using 490 nm as excitation wavelength. Data presented here are representative of three experiments. The ratio of the reading at 595 nm to the reading at 535 nm was considered as the relative ΔΨm value [19, 20].

### Analysis of ATP levels

ATP level was measured using a Cell Titer-Glo^®^ Luminescent Cell Viability Assay (Promega, Madrid, Spain), which generates a proportional signal to the ATP amount. Amoebae were incubated with different concentrations of the tested compounds for 24 hours. Aliquots were taken and mixed with the kit reagent into white plates following the manufacturer's instructions for posterior measurement of the luminescence on an Enspire Microplate Reader (Perkin Elmer Madrid, Spain) [19, 20].

## Results

### *In vitro* drug sensitivity assay

**Fig 1:**
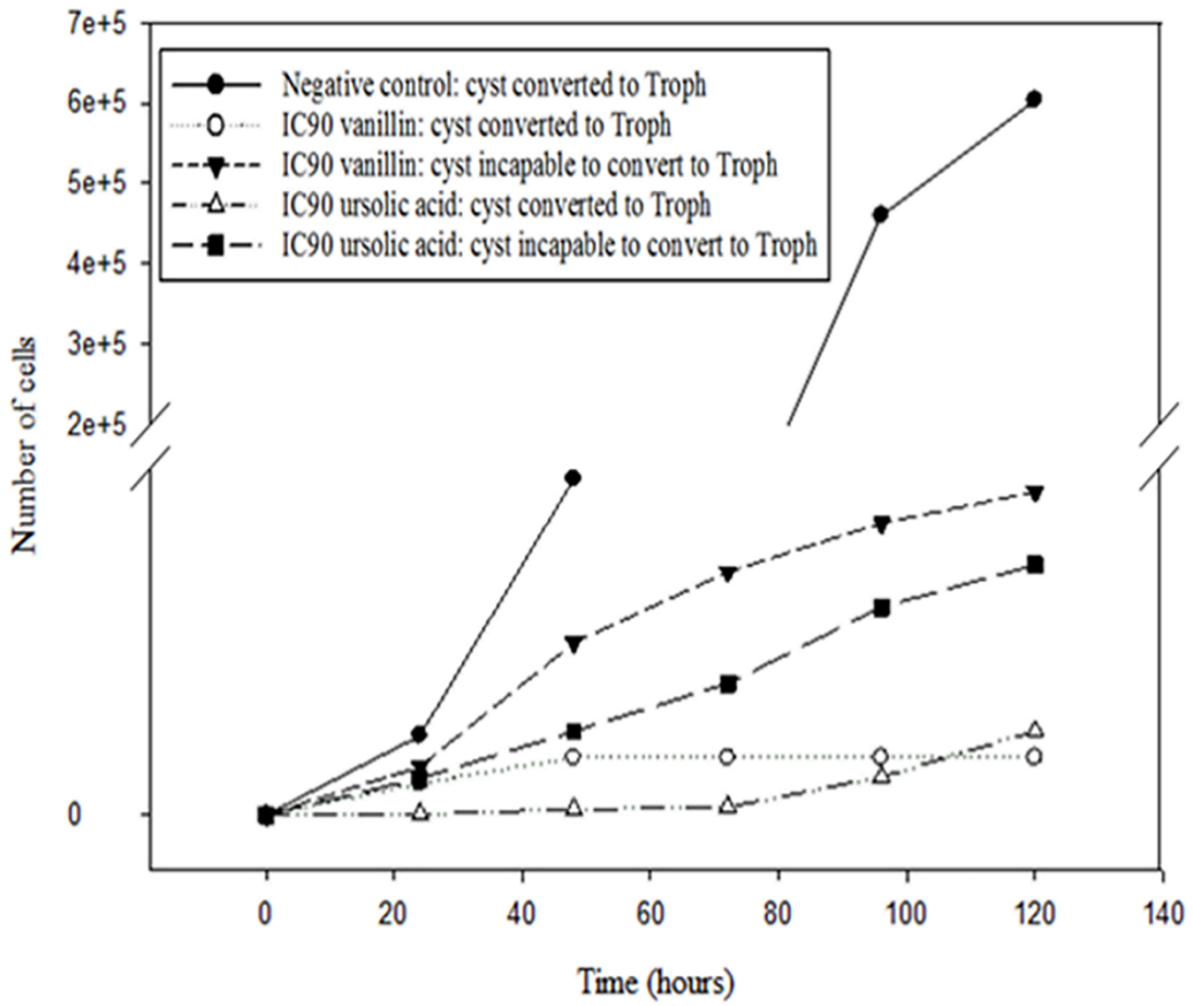
Effects of the bioactive molecules against cysts were evaluated by incubating 10^4^ cysts of *A*. *castellanii* Neff with the previously calculated IC_90_ values of the selected drugs in PYG medium, and cells were counted with a Neubauer chamber at between 96 and 168 h. Troph: Number of Trophozoite emerged from the cyst in the PYC medium.

In the present study, twenty four molecules generally present in olive leaves extracts were screened for their activity against the trophozoites stage of *Acanthamoeba castellanii* Neff. The IC_50_/96 h was chosen as the appropriate and comparable data to give as previously described [14]. The results are illustrated in Table 1. The tested parasite have been inhibited by all the tested molecules with an IC_50_ ranged from 6.59 ± 0.39 μg/ml for apigenine to an IC_50_ > 100 for 6 molecules. Based on the amoebicidal activity, vanillin, vanillic acid, syringic acid, ursolic acid, luteolin and apigenine were selected to evaluate their effect on mature cyst and macrophages J774.A1. Cysts of *A*. Neff were treated with the IC_90_ of the tested molecules. In fact, the Fig 1 illustrates that up to 120h the excystation did not occur with all the tested substances.

**Table 1:** Screening of 24 molecules against *Acanthamoeba spp* (IC_50_ and IC_90_ expressed in μg/ml).

	Molecules	*A*. Neff	CLC- 51	CLC-16
1	Gallic acid	>100	-	-
2	Vanillin	25.55 ± 2.10 IC_90_ = 123.25 ± 2.58	37.20 ± 2.85	58.11 ± 3.77
3	Caffeic acid	>100	-	-
4	Ferulic acid	62.03 ± 1.78	-	-
5	Chlorogenic acid	>100	-	-
6	*p*-coumaric acid	72.19 ± 0.19	-	-
7	m-coumaric acid	65.75 ± 3.97	-	-
8	Ellagic acid	67.36 ± 3.59	-	-
9	Vanillic acid	34.72 ± 1.32 IC_90_ = 132.65 ± 4.36	61.67 ± 4.43	65.39 ± 0.89
10	Syringic acid	22.91 ± 2.14 IC_90_ = 119.32 ± 1.65	63.07 ± 0.56	51.45 ± 2.41
11	Tyrosol	63.77 ± 0.34	-	-
12	Protochatechuic acid	36.05 ± 1.31	-	-
13	Rutin	61.36 ± 5.44	-	-
14	Oleuropein	57.52 ± 4.32	-	-
15	Hydroxytyrosol	>100	-	-
16	Luteolin	27.96 ± 1.73	26.63 ± 1.75	83.10 ± 0.89
17	Luteolin 7-o- glucoside	61.98 ± 3.93	-	-
18	Apigenine	6.59 ± 0.39	20.16 ± 0.73	56.96 ± 2.68
19	Verbascoside	>100	-	-
20	Catechin hydrate	>100	-	-
21	Quercetin hydrate	>100	-	-
22	Oleanolic acid	43.67 ± 3.34	-	-
23	Maslinic acid	30.88 ± 1.33	-	-
24	Ursolic acid	23.94 ± 1.14 IC_90_ = 119.20 ± 3.25	27.97 ± 2.26	55.02 ± 1.21

### Cytotoxicity test

**Fig 2:**
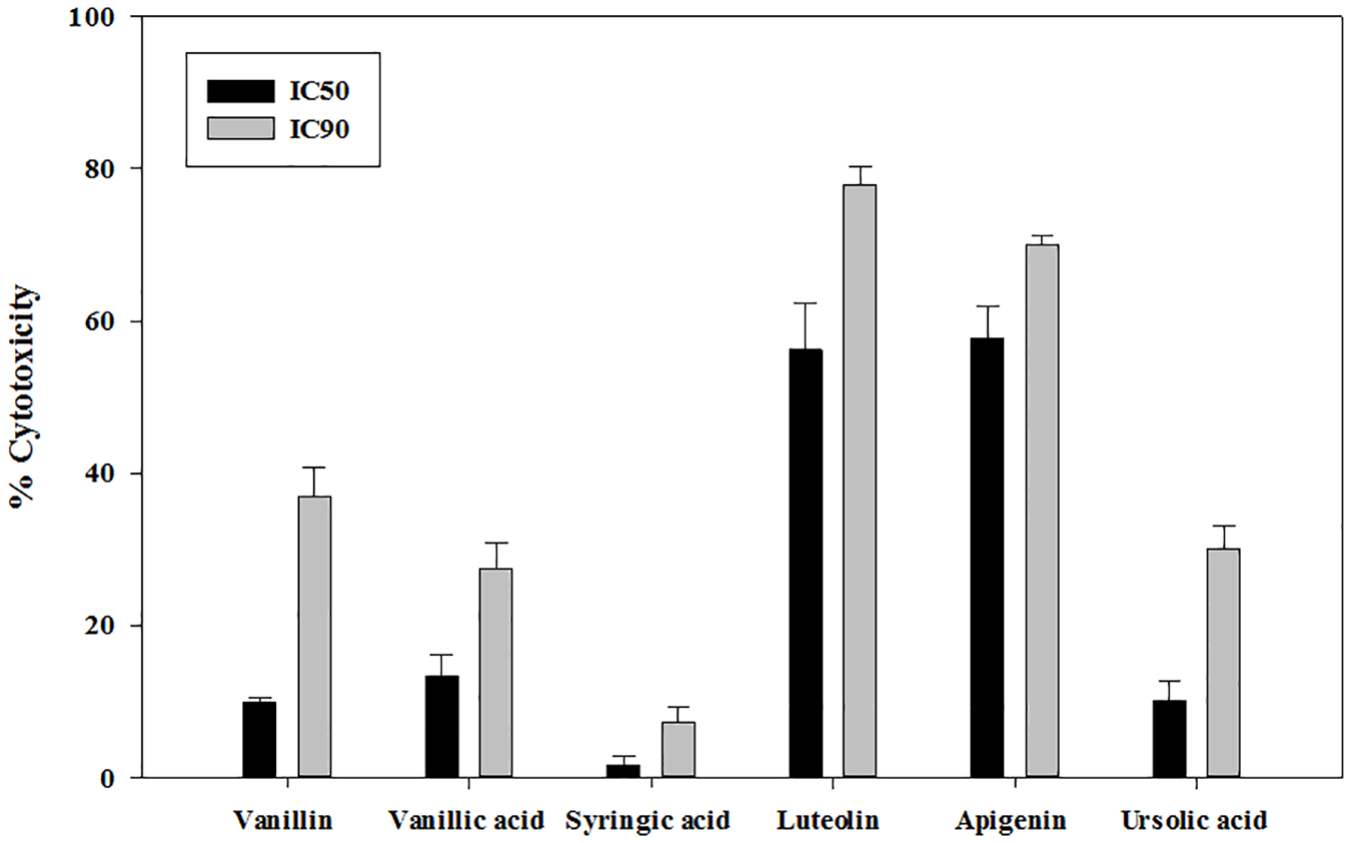
Cytotoxicity levels of the tested drugs were evaluated against murine macrophages (J774A.1) at the IC_50_ and IC_90_ against *Acanthamoeba*. Values between 10 and 25% correspond to low cytotoxicity and abve 40% correspond to high cytotoxicity. Vanillin, Vanillic acid, Syringic acid and Ursolic acid showed low cytotoxicity at the IC_50_. Syringic acid also presented low cytotoxicity at the IC_90_. Luteolin and apigenin showed high cytotoxicity (IC_50_ and IC_90_).

The histograms from the Fig 2 showed that excepting in the case of luteolin and apigenin, the other tested compounds exhibited a low to moderate cytotoxicity. The lowest toxic levels were induced by the syringic acid compared to the vanillin, vanillic and ursolic acids. Based on the cytotoxicity, four molecules were selected to characterize the action mode namely the vanillin, vanillic acid, syringic acid and ursolic acid.

### Action mode determination

**Fig 3:**
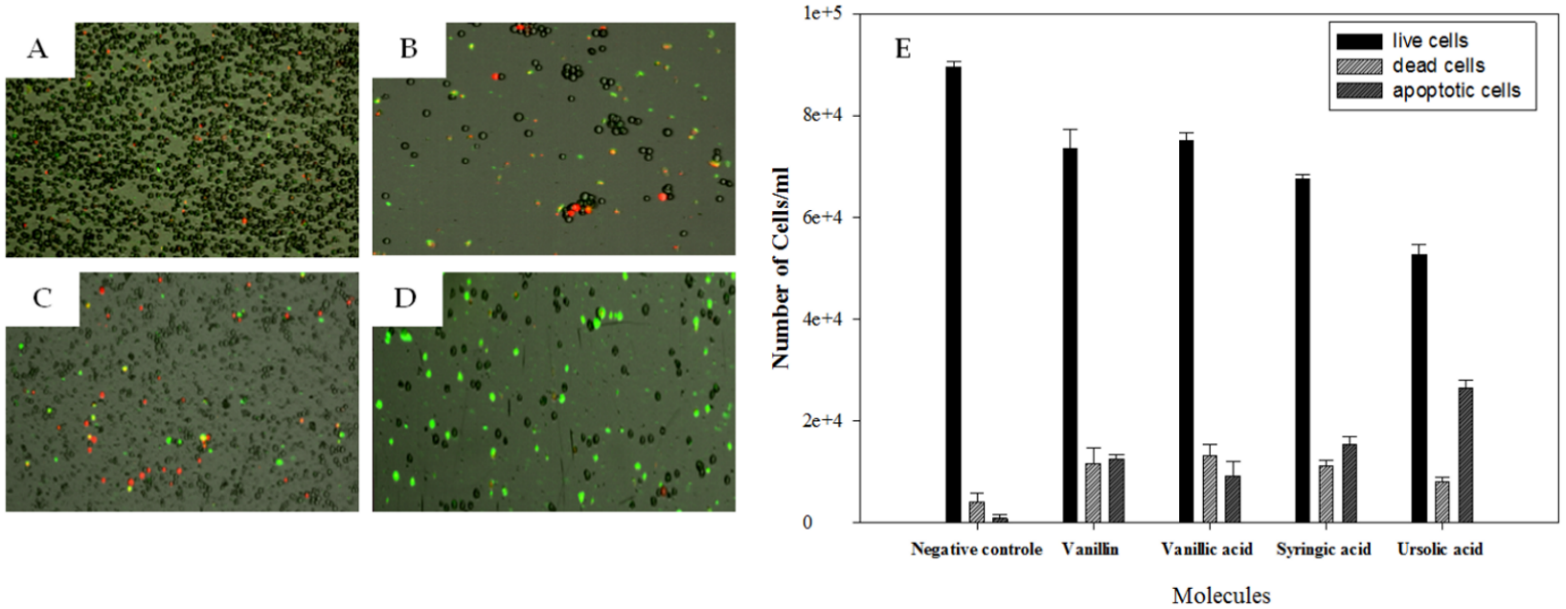
Image based Cytometer analysis for apoptosis determination (24h). Cell viability and apoptosis were evaluated with the Tali^™^ Image-based Cytometer using the Tali^™^ apoptosis kit. As a negative control we used untreated cells. Negative Control cells (A). Vanillin at IC_90_ (B), Syringic acid IC_90_ (C). Ursolic acid at IC_90_ (D). Results are represented in percentages (E).

Vanillin, vanillic, syringic and ursolic acids could induce the Programmed Cell Death in the treated cells. In order to investigate if the tested molecules induced apoptosis in *Acanthamoeba*, cells were treated with the calculated IC_90_ for each of the tested molecules and stained with the Tali^™^ Apoptosis Kit–Annexin V Alexa Fluor^®^ 488 and Propidium Iodide. As illustrated in Fig 3, the results pointed out that the ursolic acid induced the higher percentage of apoptotic cells with a percentage of 26%.

**Fig 4:**
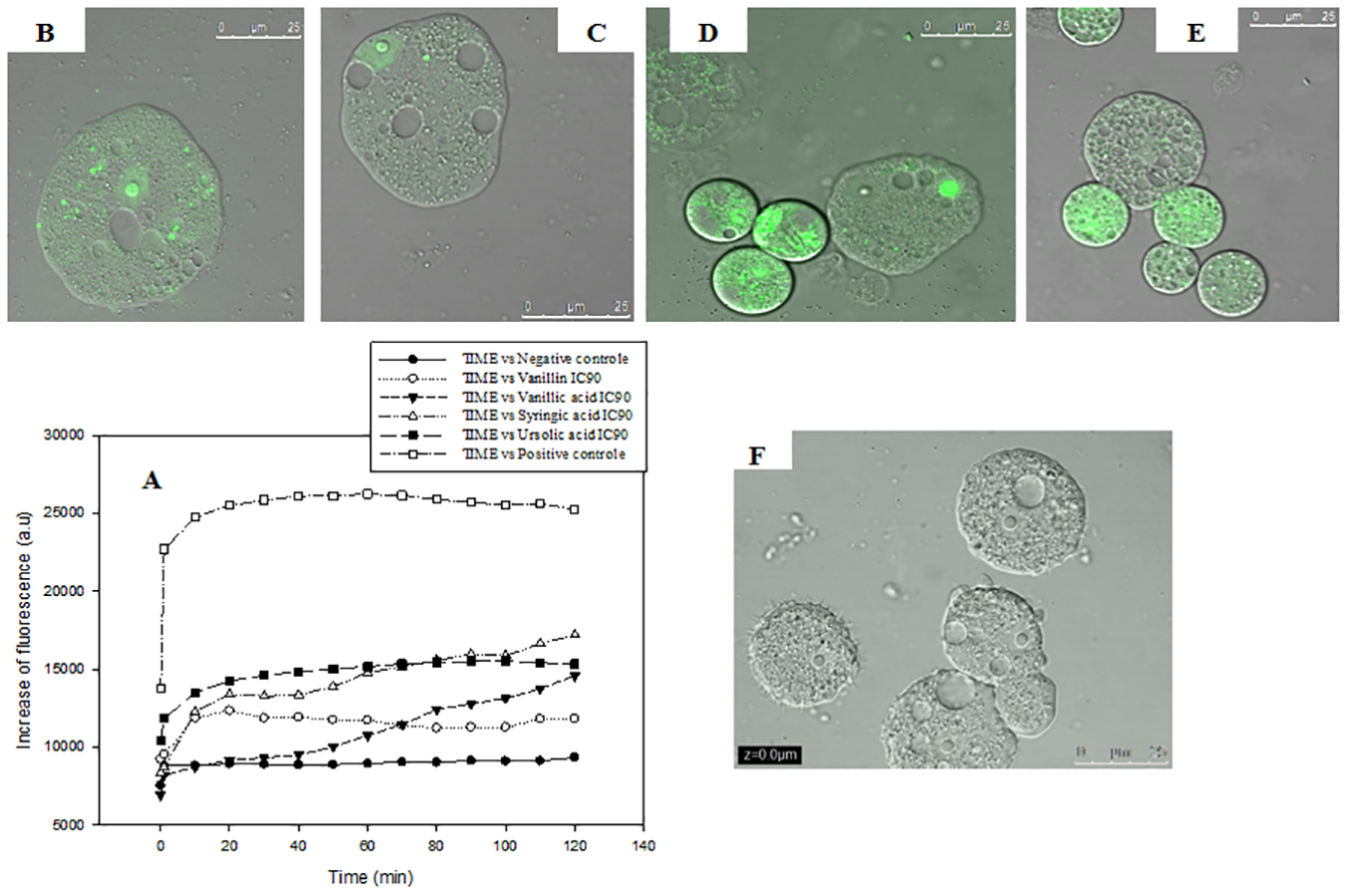
Permeation of the *Acanthamoeba* Neff to the vital dye SYTOX^®^ green caused by addition of IC_90_ of the bioactive molecules. Molecules were added to cells (10^5^ cells/ml) in the presence of 1 μM SYTOX^®^ green in PBS + 20 mM d-glucose and the increase in fluorescence (*λ*_exc_ = 485 nm, *λ*_em_ = 520 nm) monitored. Negative Control cells: cells labeled with the Sytox green in presence of 0.5% Methanol. Positive control contained 2.5% Triton X-100 (A). Confocal microscopy of *Acanthamoeba castellanii* Neff labeled with SYTOX^®^ Green. Amoeba were plated as above and incubated for 3 h with IC_90_ of the vanillin (B), vanillic acid (C) syringic acid (D) and ursolic acid (E), Negative control (F). Cells were observed in a Leica TSC SPE- confocal microscope equipped with inverted optics (*λ*_exc_ = 482 nm and *λ*_em_ = 519 nm).

Vanillin, Vanillic acid, syringic acid and ursolic acid caused plasma membrane permeability in treated cells. The possible action of the tested molecules on *A*. Neff membrane was studied using the fluorescent probe SYTOX Green. Our data clearly demonstrated that the membrane damage occurs immediately after the contact with the drugs (Fig 4). The surfactant Triton X-100 was used as a positive control to provide fully permeabilized parasites, leading to the highest fluorescence levels. Although, the membrane permeabilization was proven with the confocal microscopy and none of the tested product reached the same levels of disruption when compared to the positive control with Triton.

**Fig 5:**
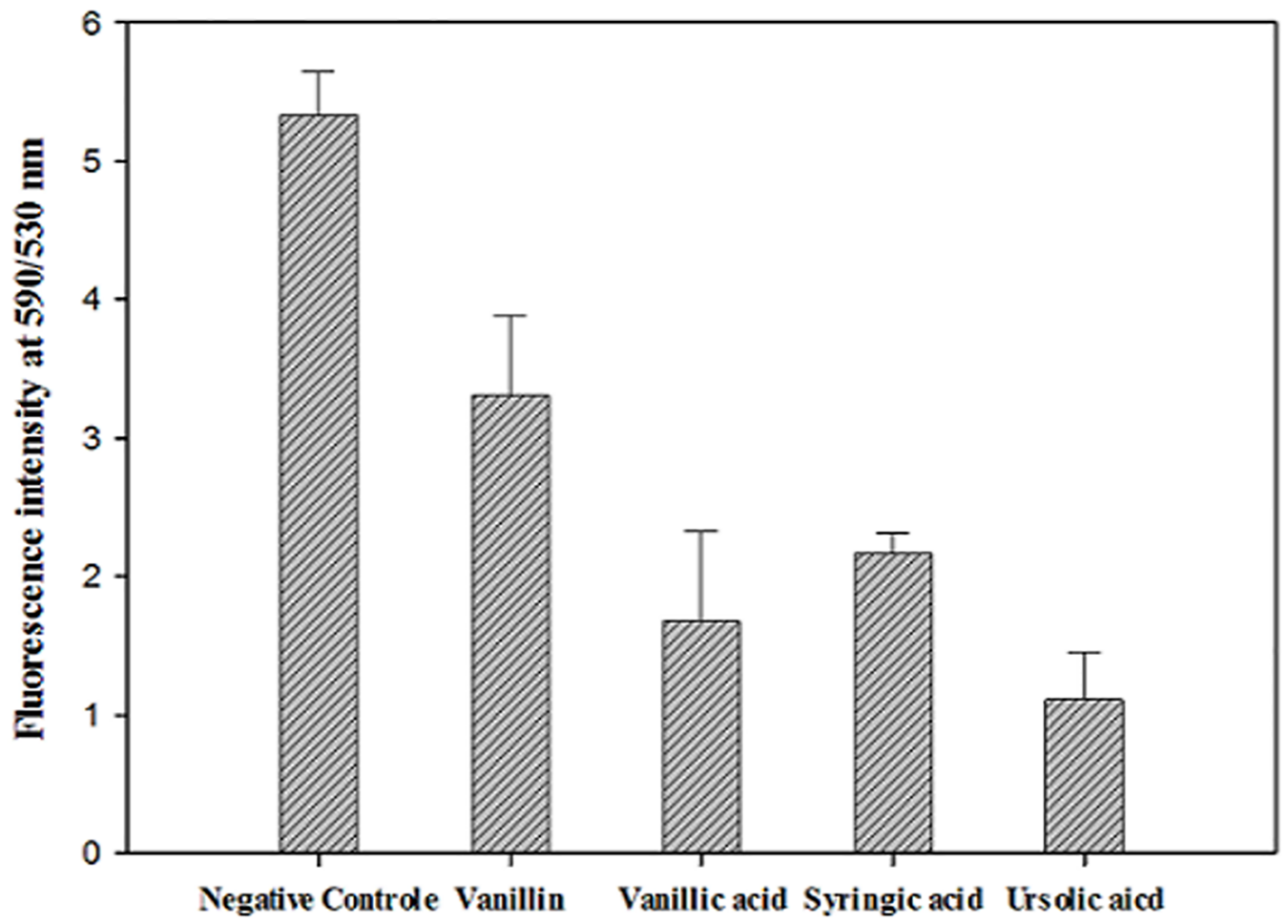
Mitochondrial membrane potential (Δψ_m_) showing change in the ratio of fluorescence intensity at 590/530 nm after the 24 hours of treatment with the IC_90_ of the all the tested molecules.

**Fig 6:**
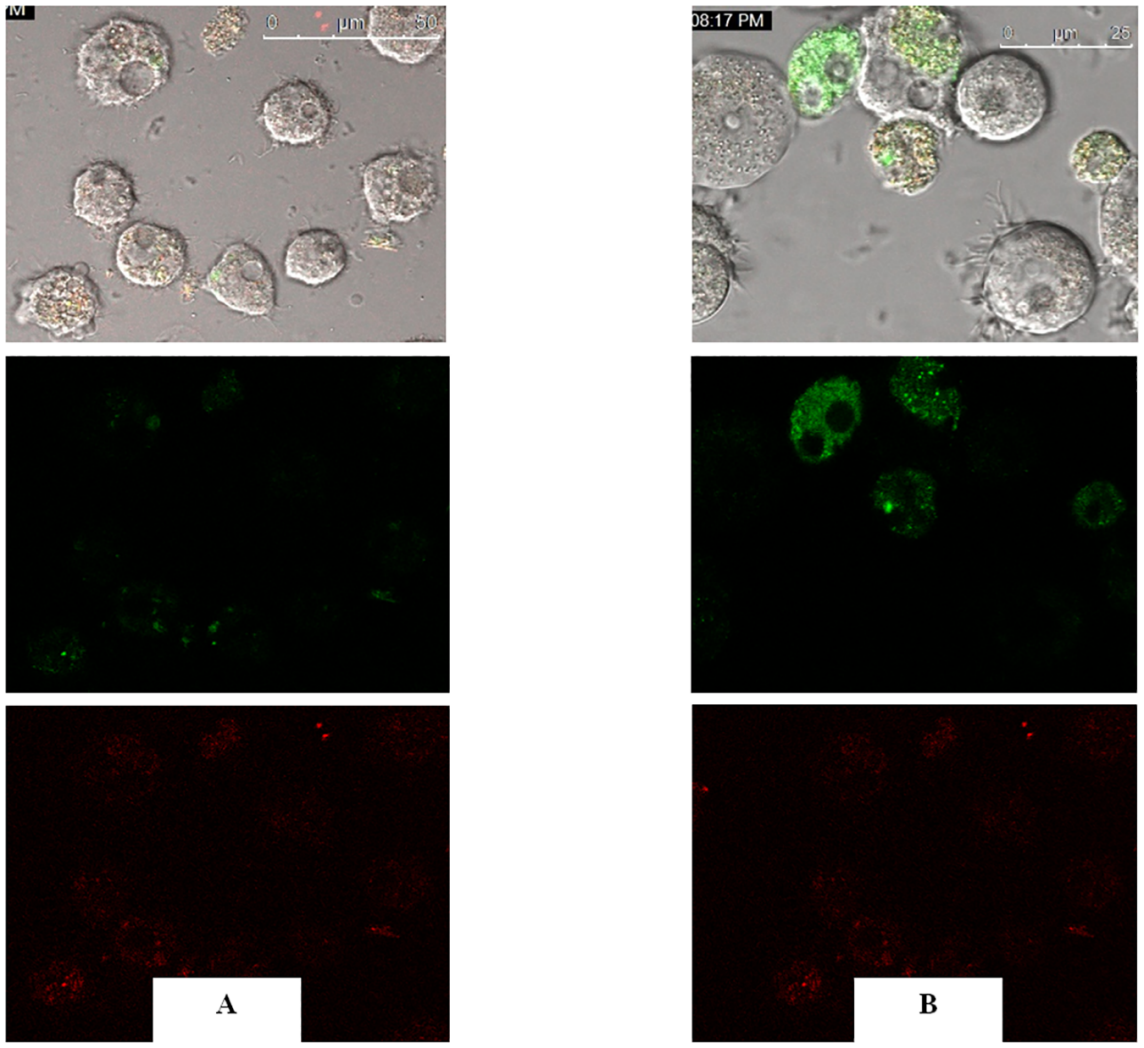
The effect of ursolic acid on the mitochondrial potential, JC-1 dye accumulates in the mitochondria of healthy cells as aggregates (red fluorescing) (Negative controle A); in cells treated with the IC_90_ of the ursolic acid for 24 h, due to collapse of mitochondrial potential, the JC-1 dye remained in the cytoplasm in its monomeric form, which fluoresced green.

**Fig 7:**
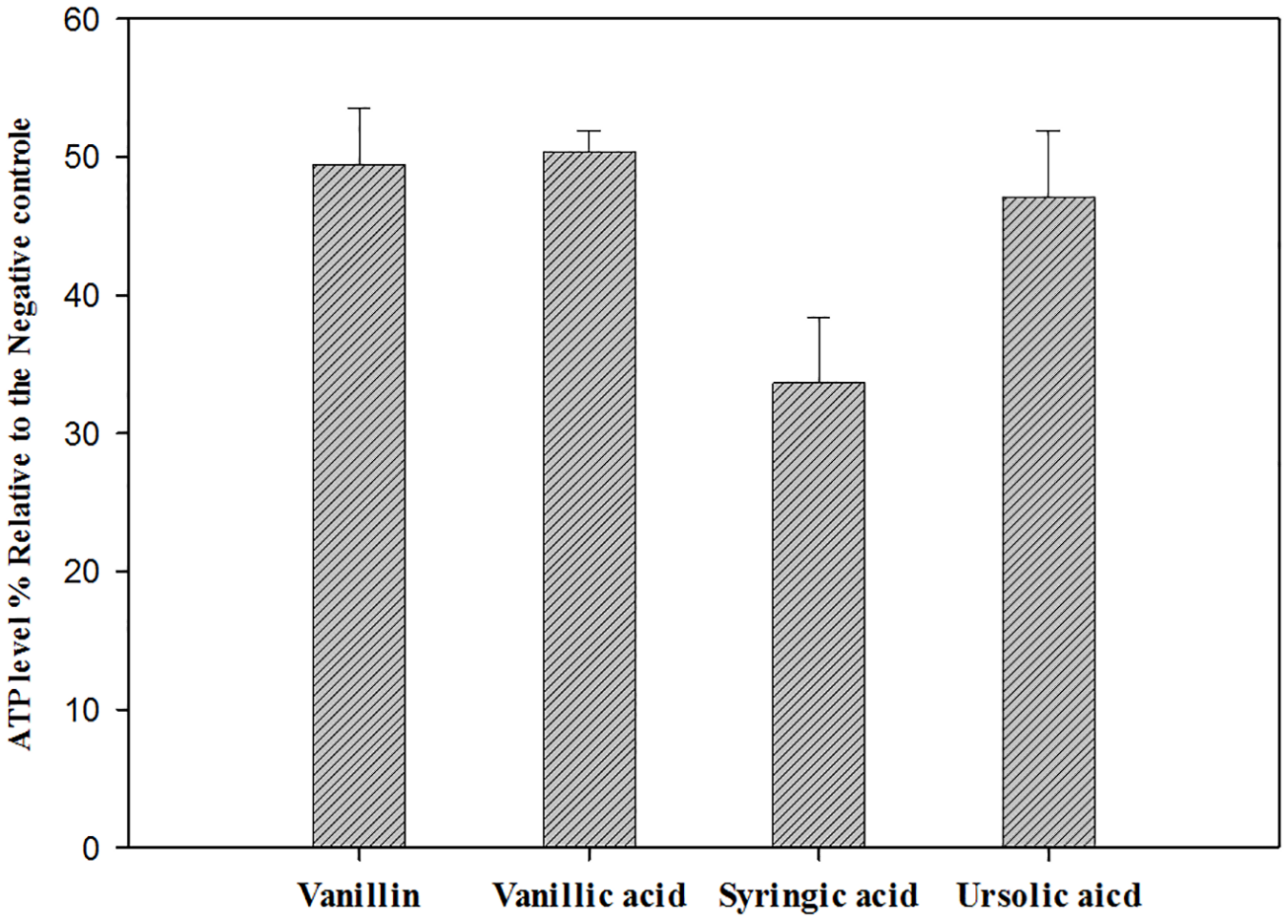
The effect of both molecules on the ATP content, using CellTiter-Glo^®^ Luminescent Cell Viability Assay. Results are representing in percentage relative to the negative control. Cells were treated by the IC_90_ concentration for 24 hours.

Vanillin, Vanillic acid, syringic acid and ursolic acid induced mitochondrial malfunction. Histograms of the mitochondrial potential fluorescence (Fig 5) demonstrated that the treatment with the IC_90_ of vanillin, vanillic acid, syringic acid and ursolic acid, decreased the membrane potential (Δ*Ψm*) of *A*. Neff by 36%, 68%, 68% and 80%, respectively. As presented in the Fig 6, confocal microscopy confirmed the effects of the ursolic acid on the mitochondrial potential. The mitochondrial damage was confirmed with the measure of ATP generation at 24h. As shown in the Fig 7, all the tested molecules dramatically decreased the levels of ATP. In fact, the cells treated with the IC_90_ of ursolic acid generated a percentage of ATP less than 50% of the untreated cells.

## Discussion

Olive leaves have been used in the pharmaceutical and cosmetic industries due to its pharmacological properties. In fact, the leaves contain several constituents responsible of those activities, including phenolic acid, secoiridoides, flavonoids, and triterpenic acids. All the tested molecules presented an antiamoebic activity although; some of them present a stronger activity with a low cytotoxicity. Vanillin is one of the simple phenols widely used in food and cosmetic industries as a flavoring agent. Furthermore, this phenolic aldehyde exhibits several biological proprieties namely the antioxidant, antimicrobial, anti-inflammatory and anti-tumoral [6]. Table 1 shows that vanillin inhibited the *Acanthamoeba* strains with an IC_50_ ranging from 25.55 ± 2.10 to 58.11 ± 3.77 μg/ml for *A*. Neff and CLC16 respectively. Phenolic acids are a group of natural products, commonly found in food, endowed with a strong antiradical activity due to the presence of hydroxyl groups [21]. As for the antiprotozoal activity, Derda et al, (2013) reported the amoebicidal activity of a phenolic acid fraction extract from *Eryngium planum* [22]. In the present study, all the tested phenolic compounds exhibited an antiamoebic activity with different IC_50_. Among them, the syringic acid showed the strongest activity with an IC_50_ of 22.91 ± 2.14. Among, the tested flavonoids, apigenin presented the highest antiamoebic activity with an IC_50_ of 6.59 ± 0.39 μg/ml. Several reports, have confirmed its therapeutic effect as anti-inflammatory, antioxidant and anticancer agents [23]. The inhibition of *Acanthamoeba* Neff growth by both oleanolic and maslinic acids have been reported in a previous work [5]. Moreover, the ursolic acid showed a stronger activity than the previous triterpenic acids with an IC_50_ of 3.94 ± 1.14 μg/ml. Due to their high activity and low to moderate cytotoxicity four molecules have been retained to accomplish the present work namely, the vanillin, syringic acid, vanillic acid, and ursolic acid.

Programmed cell death (PCD) and apoptosis-like processes have been already described in unicellular protists and in multicellular organisms [19]. This process include several morphological changes namely the chromatin condensation, nuclear DNA fragmentation, cell shrinkage, loss of mitochondrial membrane potential, the formation of apoptotic bodies, and the exposure of phosphatidylserine [24]. Among the tested drugs, the ursolic acid induced the highest percentage of apoptotic cells. Several reports confirmed the pharmacological properties of this molecule, especially as a chemo-preventive agent for cancer. In fact, Kim et al, (2011) demonstrated that ursolic acid could inhibit the proliferation of human breast cancer cell line (MDA-MB-231) and thus by inducing apoptosis [25]. Recently, in the case of *Acanthamoeba*, Martín-Navarro et al. (2015) described induction of PCD in these protozoa [17]. Furthermore, the authors demonstrated that both statins and voriconazole induced PCD related processes.

The possible mechanism of action of the tested molecules on the cell membrane was studied using the fluorescent probe SYTOX Green. Our data clearly demonstrated that the membrane damage occurs immediately after incubation with the tested molecules although; none of the tested drugs were able to reach the fluorescence level of the positive control (2.5% Triton X-100). The tested drugs affected the membrane permeability leading to the entrance of the molecules without necrotic effects [26].

The ATP generation in cells depends on the mitochondrial perturbation and especially of its membrane potential [27]. When mitochondria are affected, cellular synthesis of ATP is blocked and thus cells could lead to programmed cell death [27]. In the present study, we founded that all the tested molecules induced a pronounced decrease in the mitochondrial potential and therefore in the total ATP levels. Moreover, these events verify that the studied drugs could induce apoptosis in the *Acanthamoeba* cells through the intrinsic pathway.

## Conclusions

In conclusion, the obtained results suggest that several molecules present in the olive leaf extract possess an interesting amoebicidal activity. Nevertheless and based on the cytotoxicity effects on the macrophage cell line, four molecules have been retained namely, vanillin, vanillic, syringic and ursolic acids. Considering the observed effects on the mitochondria function by decreasing both the mitochondrial potential and the ATP content, most of the bioactive molecules seem to induce apoptosis via mitochondrial pathway. Although, further studies are necessary in order to validate this hypothesis by studying characteristic signals of this pathway such as ROS generation and DNA fragmentation.
